# Depression and its influencing factors among older adults with chronic pain in China: an empirical analysis based on CHARLS data

**DOI:** 10.3389/fpubh.2025.1547860

**Published:** 2025-04-02

**Authors:** Guojun Wang, Shiwei Huang, Ning Sun, Wenjin Gui, Yongjun Wang

**Affiliations:** ^1^Department of Public Health, Shenzhen Clinical Research Center for Mental Disorders, Shenzhen Mental Health Center/Shenzhen Kangning Hospital, Shenzhen, China; ^2^Department of Geriatric Psychiatry, Shenzhen Clinical Research Center for Mental Disorders, Shenzhen Mental Health Center/Shenzhen Kangning Hospital, Shenzhen, China; ^3^Department of Medical Treatment, Shenzhen Clinical Research Center for Mental Disorders, Shenzhen Mental Health Center/Shenzhen Kangning Hospital, Shenzhen, China; ^4^Department of Psychiatric Prevention and Treatment, Baise Second People’s Hospital, Baise, China

**Keywords:** older adult, pain, depression, influencing factor, CHARLS

## Abstract

**Background:**

China’s aging problem is intensifying, the older adult not only face a variety of chronic physical diseases and pain, but also have higher levels of depression than other age groups. This study explores the related factors of depression in older adults with chronic pain in China and provide evidence and reference for the formulation of intervention policies and measures.

**Methods:**

Using the data of the fifth wave of national survey conducted by the China Health and Retirement Longitudinal Study (CHARLS) in 2020, a total of 10,581 older adults with chronic pain were selected as research objects, and their depression status was measured by the Depression Scale (CES-D). Chi-square test and multiple logistic regression were used to analyze the main factors affecting depression in older adults with chronic pain.

**Results:**

The results of multivariate logistic regression analysis showed: gender (female: OR = 1.28, 95%CI = 1.16–1.41), age (≥75 years old: OR = 0.49, 95%CI = 0.42–0.56), spouse/partner living together (no: OR = 1.19, 95%CI = 1.06–1.32), place of residence (rural: OR = 1.19, 95%CI = 1.06–1.32), education level (High school and above: OR = 1.19, 95%CI = 1.06–1.32); satisfaction with child relationship (satisfaction: OR = 0.22, 95%CI = 0.18–0.28), smoking (no: OR = 0.60, 95%CI = 0.41–0.86), Internet use in the past month (Yes: OR = 0.77, 95%CI = 0.68–0.86), nap duration (1 ~ <2 h: OR = 0.75, 95%CI = 0.66–0.85; ≥2 h: OR = 0.75, 95%CI = 0.66–0.85), night sleep duration (6 ~ <8 h: OR = 0.75, 95%CI = 0.66–0.85; ≥8 h: OR = 0.56, 95%CI = 0.49–0.63), BADL damaged (Yes: OR = 1.45, 95%CI = 1.31–1.62), IADL damaged (Yes: OR = 1.31, 95%CI = 1.17–1.45), received outpatient services in the past month (Yes: OR = 1.18, 95%CI = 1.06–1.31), pain (Quite a Bit/Very: OR = 1.41, 95%CI = 1.26–1.58), number of body parts that feeling pain (1 ~ 3: OR = 1.42, 95%CI = 1.27–1.60; 4–6: OR = 1.76, 95%CI = 1.51–2.04; 7 ~ 9: OR = 2.21, 95%CI = 1.82–2.67; ≥10: OR = 2.63, 95%CI = 2.15–3.22) are the influencing factors of depressive symptoms in older adults with chronic pain (*p* < 0.05).

**Conclusion:**

The incidence of depressive symptoms in older adults with chronic pain is 31.7%, and their depression status is affected by various factors. Medical and health institutions and policy makers should pay attention to the mental health of these older adults, and take targeted measures to improve health education, disease treatment, pain management, sleep improvement, family support, and other aspects according to their characteristics.

## 1 Introduction

A Meta-analysis of depressive symptoms in older adults worldwide showed an overall prevalence of depressive symptoms in older adults of 28.4% (95% CI: 24.8–32.0%) between 2000 and 2021 (1). The overall prevalence of depressive symptoms in the older adult in China is lower than the global overall prevalence of depressive symptoms in the older adult. According to a recent Meta-analysis, the overall prevalence of depressive symptoms among Chinese mainland aged 60 years and older was 20.0% (95% CI: 17.5–22.8%) as January 2020 and before, estimates for mild and moderate-to-severe depressive symptoms were 16.6% (95% CI: 13.0–21.0%) and 4.5% (95% CI: 3.2–6.3%), respectively (2). Depressive symptoms not only increase the risk of dementia (3), but are also a common risk factor for suicide (4). In terms of years of disability and life lost, depression ranks first in the global burden of disease (5). According to the data of the seventh national population census in 2020, there were 260 million people aged 60 and over, accounting for 18.7% of the total population, of whom 190 million are aged 65 and over, accounting for 13.5% of the total population (6). Therefore, depression has a huge impact on the health of the older adult in China, which requires special attention.

Pain is an unpleasant subjective feeling and emotional experience related to tissue injury or potential tissue injury. The International Pain Research Institute defines chronic pain as pain that persists for more than 3 months or after the injury has healed (7). Chronic pain affects everyone in the world and has become an unavoidable problem. Chronic pain not only seriously affects the quality of sleep, but also prolonged chronic pain can also lead to depression and quality of life. The prevalence of chronic pain in China has increased year by year, affecting more and more older adults people (8). It is estimated that by 2025, 20% patients with chronic pain in China are expected to be affected (9). In view of China’s current situation, it is necessary to pay attention to the pain management of the older adults. The previous studies on chronic pain have mainly focused on the pain treatment and pathological mechanisms (10–12). Peripheral nerve stimulation (PNS) is employed to treat a wide range of chronic pain conditions (12). However, it is important to note that not all chronic pain is managed in exactly the same way. Multimodal pain management is regarded as the most helpful treatment form for chronic pain (11). Clinical practice guidelines often recommend a conservative approach for managing neck pain, which includes education, exercise therapy (ET), manual therapy (MT), and pharmacological therapy (medication), tailored to the chronicity of the condition—whether it is acute, subacute, or chronic pain (10). However, few studies have focused on depression and its influencing factors in older adults with chronic pain.

Therefore, this study draws on data from the fifth wave investigation of the China Health and Retirement Longitudinal Study (CHARLS) in 2020, accessed the CHARLS website. Older adults with chronic pain in China were selected as participants. We analyze the status of depression and its influencing factors in older adults with chronic pain in China, aiming to provide a reference for improving the mental health status of the older adult with chronic pain.

## 2 Materials and methods

### 2.1 Study design and participants

The data for this study were extracted from the fifth wave of national survey conducted by CHARLS in 2020. CHARLS is the first nationally representative population survey conducted across 23 Chinese provinces, focusing on individuals aged 45 and above. This survey follows a randomized process using a multi-stage sampling method stratified by proportional probability to size sampling (13). The purpose of this study was to examine the current situation of depression and its influencing factors in older adults with chronic pain. Older adults aged 60 and above with chronic pain were included, and those with missing values in key variables were excluded. Finally, 10,581 older adults with chronic pain were selected as research subjects.

### 2.2 Measures

#### 2.2.1 Measurement of demographic characteristics

Participants were asked about their gender, date of birth, place of residence, marital status, highest level of education, endowment insurance, medical insurance, smoking status, alcohol consumption status, satisfaction with child relationship, whether they had received outpatient services in the past month, internet use in the past month, experienced a traffic accident, or any major accident injury, whether they had ever fallen, chronic diseases, and social activities in the past month.

#### 2.2.2 Chronic pain

Participants were asked, “Do you often have troubled with body pain?” The options were “not at all,” “a little,” “some,” “quite a bit,” “very much.” If participants answered any of the “a little,” “some,” “quite a bit,” “very much” options, they were then asked, “Which parts of the body are experiencing pain? (Please list all parts).” These include headache, shoulder pain, arm pain, wrist pain, finger pain, chest pain, stomachache, back pain, waist pain, buttocks pain, leg pain, knee pain, ankle pain, toes pain, neck pain, and other pain.

#### 2.2.3 Depressive symptoms

Depression symptoms were measured using the Center for Epidemiologic Studies Depression Scale (CES-D-10), which has been validated with older adults in China. The scale includes 10 self-rated questions regarding participants’ experiences during the past week. Each question has four options: “rarely or none” is assigned 0 points, “some or a little” is assigned 1 point, “occasionally or a moderate amount of time” is assigned 2 points, “most of the time” is assigned 3 points, positive entries are assigned negative points. Total CES-D-10 scores range from 0 to 30, with higher scores indicating more serious depressive symptoms. We used a cut-off score of ≥10 to distinguish participants with depression, and score of <10 was classified as indicating no depressive symptoms (14). The CES-D-10 has shown good effectiveness and reliability in Chinese (15). In this study, Cronbach’s α for the CES-D-10 scale was 0.920, indicating good reliability, and the KMO value was 0.937, indicating good validity.

#### 2.2.4 Activities of daily living

In this study, “activities of daily living” refers to the ability of the older adults to perform daily living tasks. This concept is divided into two main components: BADL and IADL. Using data from the CHARLS, this study conducted a detailed assessment of these two types of activity capabilities. BADL involves six fundamental self-care activities: dressing, bathing or showering, eating, getting into or out of bed, using the toilet, and controlling urination and defecation. On the other hand, IADL includes six more complex household and social activities: doing household chores, cooking, shopping for groceries, making phone calls, taking medication, and managing money.

Respondents selected from four options based on their ability to perform these activities: (1) do not have any difficulty, (2) have difficulty but can still do it, (3) have difficulty and need help, (4) can not do it. By summing the scores of these options, a continuous variable ranging from 12 to 48 was derived to quantify the daily activity capability of the older adults. A higher value of this variable indicates greater difficulty encountered in daily activities, that is, poorer daily activity capability. The option “do not have any difficulty” was selected for each item in BADL and IADL is defined as no functional impairment. Otherwise, it is defined as functional impairment (16, 17).

### 2.3 Ethical approval

The current wave of CHARLS survey was approved by the Biomedical Ethics Committee of Peking University, and the field work plan of this round of household questionnaire survey was approved (approval number is IRB00001052-11015) (18).

### 2.4 Statistical analysis

The data were analyzed by R software version 4.3.2. The counting data were expressed as relative numbers, and the chi-square test was used for single-factor analysis. Then, the variables with statistical significance were included in the multivariate logistic regression model to analyze the influencing factors of depressive symptoms in patients with chronic diseases, utilizing the R packages dplyr, tableone, and broom. Two-sided values of *p* < 0.05 were considered statistically significant.

## 3 Results

### 3.1 Characteristics of study participants

Among 10,581 older adults with chronic pain, 5,119 (48.4%) were male and 5,462 (51.6%) were female; 3,307 people aged 60–64 (31.3%), 3,159 people aged 65–69 (29.9%), 1,936 people aged 70–74 (18.3%), 2,179 people aged 75 and over (20.6%); 3,612 people (34.1%) lived in urban areas and 6,969 people (65.9%) lived in rural areas. There were 8,070 people (76.3%) living with spouse/partner, and 2,511 people (23.7%) living without spouse/partner; 8,216 (77.6%) had elementary school education and below, 1,479 (14.0%) had middle school education, and 886 (8.4%) had high school education and above.

### 3.2 Status of chronic pain and depression in older adults with chronic pain

Of the 10,581 older adults with chronic pain, 7,230 (68.3%) had a little/somewhat pain, and 3,351 (31.7%) had quite a bit/very pain; 6,693 people (63.3%) had no depressive symptoms and 3,888 people (36.7%) had depressive symptoms, as shown in Table 1. The comparison of depression symptoms in the older adults with chronic pain in different body parts is shown in Table 2. There are statistical differences between the pain in various parts of the body and the number of body parts that feeling pain and depression in the older adults.

**Table 1:** Chronic pain and depression in older adults with chronic pain in body parts.

Variable	*N*	%
Chronic pain		
A little/somewhat	7,230	68.3
Quite a bit/very	3,351	31.7
Depression		
No	6,693	63.3
Yes	3,888	36.7

**Table 2:** Comparison of depressive symptoms in the older adults with chronic pain in different body parts.

Variable		No depression	Depression	Chi-square value	*p*
		*N* (%)	*N* (%)		
Headache	No	5,710 (85.3)	2,646 (68.1)	<0.001	441.06
	Yes	983 (14.7)	1,242 (31.9)		
Shoulder pain	No	5,646 (84.4)	2,686 (69.1)	<0.001	342.73
	Yes	1,047 (15.6)	1,202 (30.9)		
Arm pain	No	5,920 (88.5)	2,905 (74.7)	<0.001	335.12
	Yes	773 (11.5)	983 (25.3)		
Wrist pain	No	6,203 (92.7)	3,241 (83.4)	<0.001	222.73
	Yes	490 (7.3)	647 (16.6)		
Fingers pain	No	6,119 (91.4)	3,108 (79.9)	<0.001	290.74
	Yes	574 (8.6)	780 (20.1)		
Chest pain	No	6,266 (93.6)	3,282 (84.4)	<0.001	236.63
	Yes	427 (6.4)	606 (15.6)		
Stomachache	No	6,023 (90.0)	3,041 (78.2)	<0.001	277.62
	Yes	670 (10.0)	847 (21.8)		
Back pain	No	5,972 (89.2)	2,979 (76.6)	<0.001	299.95
	Yes	721 (10.8)	909 (23.4)		
Waist pain	No	4,934 (73.7)	2,082 (53.5)	<0.001	447.83
	Yes	1,759 (26.3)	1,806 (46.5)		
Buttocks pain	No	6,256 (93.5)	3,301 (84.9)	<0.001	206.57
	Yes	437 (6.5)	587 (15.1)		
Leg pain	No	5,346 (79.9)	2,413 (62.1)	<0.001	398.95
	Yes	1,347 (20.1)	1,475 (37.9)		
Knees pain	No	5,242 (78.3)	2,352 (60.5)	<0.001	385.76
	Yes	1,451 (21.7)	1,536 (39.5)		
Ankle pain	No	6,107 (91.2)	3,115 (80.1)	<0.001	271.97
	Yes	586 (8.8)	773 (19.9)		
Toes pain	No	6,282 (93.9)	3,321 (85.4)	<0.001	208.97
	Yes	411 (6.1)	567 (14.6)		
Neck pain	No	6,028 (90.1)	3,031 (78.0)	<0.001	292.69
	Yes	665 (9.9)	857 (22.0)		
Other pain	No	6,272 (93.7)	3,462 (89.0)	<0.001	72.729
	Yes	421 (6.3)	426 (11.0)		
Number of body parts that feeling pain	0	3,274 (48.9)	1,000 (25.7)	<0.001	826.93
	1–3	2,113 (31.6)	1,281 (32.9)		
	4–6	732 (10.9)	679 (17.5)		
	7–9	316 (4.7)	419 (10.8)		

### 3.3 Univariate analysis of depression in older adults with chronic pain

The prevalence of high-risk depression significantly differed among the groups in terms of gender, age, spouse/partner living together, place of residence, education level, medical insurance, nap duration, night sleep duration, smoking, alcohol consumption, satisfaction with child relationship, received outpatient service in the past month, internet use in the past month, experienced a traffic accident, or any major accident injury, ever falling down, chronic pain, number of social activities in the past month, BADL damaged, IADL damaged and number of chronic diseases. There was no statistically significant difference in the prevalence of depressive symptoms in older chronic pain patients with different pension insurance, as shown in Table 3.

**Table 3:** Univariate analysis of depressive status in older adults with chronic pain.

Variable		No depression	Depression	Chi-square value	*p*
		*N* (%)	*N* (%)		
Gender	Male	3,599 (53.8)	1,520 (39.1)	212.16	<0.001
	Female	3,094 (46.2)	2,368 (60.9)		
Age (year)	60–64	2,095 (31.3)	1,212 (31.2)	54.93	<0.001
	65–69	1,942 (29.0)	1,217 (31.3)		
	70–74	1,143 (17.1)	793 (20.4)		
	> = 75	1,513 (22.6)	666 (17.1)		
Spouse/partner living together	No	5,213 (77.9)	2,857 (73.5)	26.36	<0.001
	Yes	1,480 (22.1)	1,031 (26.5)		
Place of residence	Urban	2,573 (38.4)	1,039 (26.7)	150.25	<0.001
	Rural	4,120 (61.6)	2,849 (73.3)		
Education level	Elementary school and above	4,980 (74.4)	3,236 (83.2)	125.55	<0.001
	Middle school	1,027 (15.3)	452 (11.6)		
	High school and above	686 (10.2)	200 (5.1)		
Endowment insurance	No	902 (13.5)	556 (14.3)	1.40	0.248
	Yes	5,791 (86.5)	3,332 (85.7)		
Medical insurance	No	332 (5.0)	238 (6.1)	6.50	0.012
	Yes	6,361 (95.0)	3,650 (93.9)		
Nap duration	<1 h	2,085 (31.2)	1,477 (38.0)	52.69	<0.001
	1 ~ <2 h	1,343 (20.1)	672 (17.3)		
	> = 2 h	3,265 (48.8)	1,739 (44.7)		
Night sleep duration	<6 h	2,602 (38.9)	2,247 (57.8)	357.21	<0.001
	6 ~ <8 h	2,665 (39.8)	1,109 (28.5)		
	> = 8 h	1,426 (21.3)	532 (13.7)		
Smoking status	No	6,550 (97.9)	3,843 (98.8)	13.51	<0.001
	Yes	143 (2.1)	45 (1.2)		
Alcohol consumption status	No	4,342 (64.9)	2,832 (72.8)	71.48	<0.001
	Yes	2,351 (35.1)	1,056 (27.2)		
Satisfaction with child relationship	Dissatisfied/No children	137 (2.0)	404 (10.4)	352.93	<0.001
	Satisfied	6,556 (98.0)	3,484 (89.6)		
Received outpatient services in the past month	No	5,426 (81.1)	2,842 (73.1)	91.53	<0.001
	Yes	1,267 (18.9)	1,046 (26.9)		
Internet use in the past month	No	5,066 (75.7)	3,260 (83.8)	97.57	<0.001
	Yes	1,627 (24.3)	628 (16.2)		
Experienced a traffic accident, or any major accident injury	No	6,535 (97.6)	3,767 (96.9)	5.41	0.024
	Yes	158 (2.4)	121 (3.1)		
Ever fall down	No	5,557 (83.0)	2,875 (73.9)	125.32	<0.001
	Yes	1,136 (17.0)	1,013 (26.1)		
Chronic pain	A little/somewhat	5,158 (77.1)	2,072 (53.3)	642.31	<0.001
	Quite a bit/very	1,535 (22.9)	1,816 (46.7)		
Number of social activities in the past month	0	3,745 (56.0)	2,206 (56.7)	7.71	0.021
	1	1,844 (27.6)	1,118 (28.8)		
	≥2	1,104 (16.5)	564 (14.5)		
BADL damaged	No	5,173 (77.3)	2,232 (57.4)	462.81	<0.001
	Yes	1,520 (22.7)	1,656 (42.6)		
IADL damaged	No	4,882 (72.9)	2,133 (54.9)	359.83	<0.001
	Yes	1,811 (27.1)	1,755 (45.1)		
Number of chronic diseases	0	4,071 (60.8)	2,107 (54.2)	54.55	<0.001
	1–2	2,276 (34.0)	1,486 (38.2)		
	≥3	346 (5.2)	295 (7.6)		

### 3.4 Multivariate logistic regression analysis of depression in older adults with chronic pain

Whether older adults with chronic pain had depressive symptoms was the dependent variable (assigned: No depressive symptoms =0, depressive symptoms =1). Variables with statistically significant differences in the above univariate analysis were included as independent variables, and multivariate logistic regression analysis was performed. The results showed that the influencing factors of depression in older adults with chronic pain were gender, age, spouse/partner living together, place of residence, education level, nap duration, night sleep duration, satisfaction with child relationship, smoking, received outpatient services in the past month, internet use in the past month, chronic pain, BADL damaged, IADL damaged and number of body parts that feeling pain (*p* < 0.05), as shown in Table 4.

**Table 4:** Multivariate logistic regression analysis of depression status in older adults with chronic pain.

Variable		*b*	SE	*Z*	OR [95%CI]	*p*
Gender	Male					
	Female	0.25	0.05	4.88	1.28 [1.16, 1.41]	<0.001
Age (year)	60–64					
	65–69	−0.07	0.06	−1.15	0.94 [0.84, 1.05]	0.252
	70–74	−0.05	0.07	−0.81	0.95 [0.83, 1.08]	0.416
	> = 75	−0.72	0.07	−10.10	0.49 [0.42, 0.56]	<0.001
Spouse/partner living together	Yes					
	No	0.17	0.06	3.09	1.19 [1.06, 1.32]	0.002
Place of residence	Urban					
	Rural	0.34	0.05	6.81	1.41 [1.28, 1.56]	<0.001
Education level	Elementary school and above					
	Middle school	−0.02	0.07	−0.31	0.98 [0.85, 1.12]	0.754
	High school and above	−0.27	0.09	−2.84	0.76 [0.63, 0.92]	0.005
Nap duration	<1 h					
	1 ~ <2 h	−0.29	0.06	−4.52	0.75 [0.66, 0.85]	<0.001
	> = 2 h	−0.10	0.05	−1.97	0.91 [0.82, 1.00]	0.049
Night sleep duration	<6 h					
	6 ~ <8 h	−0.47	0.05	−9.32	0.63 [0.57, 0.69]	<0.001
	> = 8 h	−0.58	0.06	−9.18	0.56 [0.49, 0.63]	<0.001
Satisfaction with child relationship	No					
	Yes	−1.50	0.11	−13.83	0.22 [0.18, 0.28]	<0.001
Smoking status	No					
	Yes	−0.51	0.19	−2.71	0.60 [0.41, 0.86]	0.007
Received outpatient services in the past month	No					
	Yes	0.17	0.05	3.10	1.18 [1.06, 1.31]	0.002
Internet use in the past month	No					
	Yes	−0.27	0.06	−4.28	0.77 [0.68, 0.86]	<0.001
Chronic pain	A little/somewhat					
	Quite a bit/very	0.34	0.06	5.92	1.41 [1.26, 1.58]	<0.001
BADL damaged	No					
	Yes	0.37	0.05	6.87	1.45 [1.31, 1.62]	<0.001
IADL damaged	No					
	Yes	0.27	0.05	4.96	1.31 [1.17, 1.45]	<0.001
Number of body parts that feeling pain	0					
	1 ~ 3	0.35	0.06	5.98	1.42 [1.27, 1.60]	<0.001
	4 ~ 6	0.56	0.08	7.42	1.76 [1.51, 2.04]	<0.001
	7 ~ 9	0.79	0.10	8.18	2.21 [1.82, 2.67]	<0.001
	≥10	0.97	0.10	9.40	2.63 [2.15, 3.22]	<0.001

## 4 Discussion

This study is based on data from the fifth round of the CHARLS National Survey conducted in 2020. One major finding is that the incidence of depressive symptoms in older patients with chronic pain was 36.7% (3,888/10,581). The incidence of depressive symptoms is lower than that in older adults with chronic diseases reported by Wei Xuan (40.7%) (19), and higher than that reported by Rong Jian in the meta-analysis of the prevalence of depression in older adults in China from 2010 to 2019, which found a combined prevalence of 25.6% (20). The depression symptoms of older adults with chronic pain in China are more severe than those of the general population, highlighting the need for increased attention to the emotional well-being of this group.

Moreover, the results of multivariate logistic regression analysis in this study show that women have a higher risk of depressive symptoms than men. This is consistent with the study of Wang Yue et al. (21). This may be due to the lower family status of older women, their greater contribution to family labor and child-rearing, and the greater likelihood of widowhood and living alone (22–24). The risk of depressive symptoms is lower in those with a high school education or higher. This is consistent with the study of Song Qiuyue et al. (25). This may be due to the broader knowledge base and stronger psychological adjustment ability of highly educated older individuals. Older adults with chronic pain living in rural areas also have a higher risk of depressive symptoms. This is consistent with the study of Wu Zhengyu et al. (24). This may be due to the poor economic status, poor health, lack of social support, and lower levels of social participation among older adults with chronic pain in rural areas (26). The incidence of depressive symptoms in older adults with chronic pain aged 75 years and above is 0.49 times that of those aged 60–64 years (95%CI:0.42–0.56). This contrasts with the research results of Wang Yue et al. (21). This may be due to the fact that the study included a smaller proportion of older individuals in relatively good health.

Interestingly, we found that older adults without spouses had a higher risk of depressive symptoms. This is consistent with the study of Wang Yue et al. (21). Spouses play an important role in life, and older people without spouses may lack companionship, emotional support, financial support and relief from negative emotions. People who are more satisfied with their relationships with their children have a lower risk of depressive symptoms. This is consistent with the study of Wang Hongyu et al. (27). The older adults with high satisfaction in the relationship with their children may receive more social support and fewer conflicts from their children, which can enhance the sense of belonging and acquisition of the older adults, reduce negative emotions, and reduce the likelihood of depression in the older adults.

The findings of the study also indicate that the older adults with chronic pain who had received medical services in the past month have a higher risk of depressive symptoms. This is consistent with the research by Xu Jinpeng et al. (28). This may be due to older individuals having health problems, which can lead to more depression and greater reliance on medical care for health issues. People with severe pain had a higher risk of depressive symptoms. This is consistent with the studies by Stephen J Gibson (29) and Hiroya Honda (30). It could have been that older patients with chronic pain had poorer health, physical disabilities, or limited ability to perform daily activities.

A novel finding of this study is that the risk of depressive symptoms is higher in those with impaired BADL and IADL. This is consistent with the findings of Haixia Liu et al. (31), who found that older individuals with BADL restrictions have a higher risk of depression than those with IADL restrictions. It may be that IADL restriction precedes BADL restriction in the older adults, with IADL restriction being associated with a decreased ability to perform activities of daily living, while BADL restriction is linked to the loss of physical function, which leads to more severe negative emotions. The risk of depressive symptoms is lower in non-smokers. This is consistent with the study by Zhaoping Wu (32). This may be related to the fact that smoking increases the risk of diabetes, kidney failure, heart failure, coronary heart disease, emphysema, chronic bronchitis, cancer or malignancy, all of which, in turn, increase the risk of depression. Internet users exhibit a lower risk of depressive symptoms. This is consistent with the findings of Shelia R Cotten (33), which showed that using the Internet can reduce the likelihood of depressive states by about 33%. It may be that Internet access increases opportunities for older individuals to socialize and reduces loneliness (34).

The findings of the study also indicate that the risk of depressive symptoms is lower for nap durations of 1–<2 h and ≥ 2 h compared to naps <1 h. Similarly, the risk of depressive symptoms is lower for night sleep durations of 6–<8 h and ≥ 8 h compared to sleep durations <6 h. These findings are inconsistent with studies by Spencer A. Nielson (35) and Yanliqing Song (36). This discrepancy may be due to poor sleep quality among older individuals with chronic pain, where longer nap durations and later bedtimes help alleviate fatigue and reduce the risk of depression.

## 5 Limitations

Although the results of this study are consistent with previous research, there are several limitations. Firstly, the data were obtained from the 2020 China Health and Retirement Longitudinal Study (CHARLS), a cross-sectional study that captures the pain status of older at a single point in time. While the researchers adjusted for multiple factors, the model did not include certain variables that could influence physical pain, such as genetic factors, which will require further investigation. Secondly, since the data were derived from self-reported surveys, respondent subjectivity may affect the accuracy of the findings. Finally, the study involves a large proportion of older adults who may have provided biased responses in their questionnaires. However, these limitations are mitigated by the fact that the data represent a comprehensive, nationally representative sample drawn from a large, long-established database, which has been used for secondary data analysis of demographic characteristics, health status, and healthcare utilization among older adults with chronic pain.

## 6 Conclusion

The depression status of older adults with chronic pain is affected by gender, age, marital status, place of residence, educational level, relationship satisfaction with children, smoking, Internet use, nap duration, night sleep duration, BADL, IADL, medical service received in the past month, chronic pain and other factors. People should pay attention to their physical condition, psychological condition, interpersonal condition, self-care ability and daily living ability, and take targeted measures to improve depression. At the individual level, it is necessary to develop good behavior habits, such as not smoking, taking a nap, ensuring adequate sleep at night, and surfing the Internet appropriately. Simultaneously, the emphasis is on improving the mental health knowledge of the older adults, which, in turn, enhances their mental health literacy and self-regulation skills. At the family level, it is necessary to create a good, non-violent communication environment and family interpersonal relations, accompany the older adults more, care about the physical and psychological conditions of the older adults, and provide emotional and material support. At the community level, efforts should be made to promote the full coverage and normalization of mental care projects for the older adults in all communities across the country. It is also imperative to improve the professional level of mental care workers for older adult, and to strengthen health education on mental health knowledge and screening for mental health problems. Efforts to increase full coverage and normalization of mental care should also include timely referral, recommendation and intervention for the older adults who qualify for screening.

## Data Availability

Publicly available datasets were analyzed in this study. This data can be found at: https://charls.charlsdata.com.
